# Pathogenesis of cardiac ischemia reperfusion injury is associated with CK2α-disturbed mitochondrial homeostasis via suppression of FUNDC1-related mitophagy

**DOI:** 10.1038/s41418-018-0086-7

**Published:** 2018-03-14

**Authors:** Hao Zhou, Pingjun Zhu, Jin Wang, Hong Zhu, Jun Ren, Yundai Chen

**Affiliations:** 1 Chinese PLA General Hospital, Medical School of Chinese PLA, Beijing, China; 2 Center for Cardiovascular Research and Alternative Medicine, University of Wyoming College of Health Sciences, Laramie, WY 82071 USA

## Abstract

Disturbed mitochondrial homeostasis contributes to the pathogenesis of cardiac ischemia reperfusion (IR) injury, although the underlying mechanism remains elusive. Here, we demonstrated that casein kinase 2α (CK2α) was upregulated following acute cardiac IR injury. Increased CK2α was shown to be instrumental to mitochondrial damage, cardiomyocyte death, infarction area expansion and cardiac dysfunction, whereas cardiac-specific CK2α knockout (CK2α^*CKO*^) mice were protected against IR injury and mitochondrial damage. Functional assay indicated that CK2α enhanced the phosphorylation (inactivation) of FUN14 domain containing 1 (FUNDC1) via post-transcriptional modification at Ser13, thus effectively inhibiting mitophagy. Defective mitophagy failed to remove damaged mitochondria induced by IR injury, resulting in mitochondrial genome collapse, electron transport chain complex (ETC) inhibition, mitochondrial biogenesis arrest, cardiolipin oxidation, oxidative stress, mPTP opening, mitochondrial debris accumulation and eventually mitochondrial apoptosis. In contrast, loss of CK2α reversed the FUNDC1-mediated mitophagy, providing a survival advantage to myocardial tissue following IR stress. Interestingly, mice deficient in both CK2α and FUNDC1 failed to show protection against IR injury and mitochondrial damage through a mechanism possible attributed to lack of mitophagy. Taken together, our results confirmed that CK2α serves as a negative regulator of mitochondrial homeostasis via suppression of FUNDC1-required mitophagy, favoring the development of cardiac IR injury.

## Introduction

One of the hallmarks of cardiac ischemia reperfusion (IR) injury is the altered architecture and function of mitochondria [1]. In-depth researches from a number of laboratories have validated the importance of myocardial death following mitochondrial damage as the essential molecular basis of cardiac IR injury [2, 3]. Recently, accumulating evidence has depicted an indispensable role of mitophagy in mitochondrial protection [4]. Mitophagy, the mitochondrial-specific analog to autophagy, is considered a beneficial metabolic event and is pivotal to the preservation of mitochondrial quality [5]. Evidence from our group has shown that enhanced mitophagy alleviates mitochondrial oxidative stress, preserves mitochondrial respiratory function, limits mitochondrial debris formation, and blocks caspase 9-related apoptosis [3,6–9]. Accordingly, mitophagy is thus proposed to be a mechanistic requirement of cellular survival in cardiac IR injury and could be a promising target to relieve the IR attack [10].

Mitophagy is regulated through two distinct signaling pathways including receptor-mediated mitophagy and Parkin-dependent mitophagy [11]. The receptor-related components include BCL2/adenovirus E1B 19 kDa protein-interacting protein 3 (Bnip3) and FUN14 domain containing 1 (FUNDC1) [12]. Among which, Bnip3 is a mitochondrial outer membrane protein with biphasic effects [13], first the promotion of mitophagy and later the facilitation of cardiomyocyte death [9]. Similarly, Parkin-related mitophagy also exacerbates cardiomyocyte death in IR injury [14, 15]. On the other hand, FUNDC1-mediated mitophagy is mainly activated early by hypoxia in order to repair damaged mitochondria and offers a survival advantage for cardiomyocytes in IR injury [16, 17]. It is believed that FUNDC1-mediated mitophagy functions in a manner reminiscent of ischemic preconditioning. To this end, different mitophagy adaptors may have disparate impacts on cell fate, and that FUNDC1 may serve as the key mediator turning on protective mitophagy. Structurally, FUNDC1 has two essential phosphorylation sites including Tyr18 and Ser13. Phosphorylated FUNDC1 generates steric hindrance for LC3II binding [18], thus effectively inhibiting mitophagy [19]. Ischemic or hypoxic stimulus alleviates FUNDC1 phosphorylation at Tyr18, leading to induction of mitophagy in ischemia [16, 18]. Unfortunately, based on recent findings from our lab and others [3, 16, 20], FUNDC1 activity is downregulated in reperfusion. Nonetheless, the underlying mechanism(s) for FUNDC1 hyporeactivity during reperfusion is not completely understood. Whether reperfusion re-instates the phosphorylated inactivation of FUNDC1 and if so, the site of phosphorylation involved in reperfusion-mediated FUNDC1 inhibition remains unknown.

Casein kinase 2α (CK2α), a constitutive Ser/Thr kinase [21], was originally described as a suppressor of FUNDC1 and is linked to FUNDC1 phosphorylation at Ser13 [22]. Solid evidence has illustrated that CK2α is implicated in the development of diabetic cardiomyopathy [23] and heart failure [24]. What remains unclear is if CK2α contributes to the progression of cardiac IR injury and, if so, whether this is governed by CK2α-elicited mitochondrial injury and cardiomyocyte damage through inhibition of mitophagy mediated via FUNDC1 phosphorylation at Ser13 during reperfusion. Herein, this study aims to explore the role of CK2α and FUNDC1-required mitophagy in the development of cardiac IR injury.

## Results

### CK2α is activated by cardiac IR stimulation and promotes the development of IR injury

**Fig. 1:**
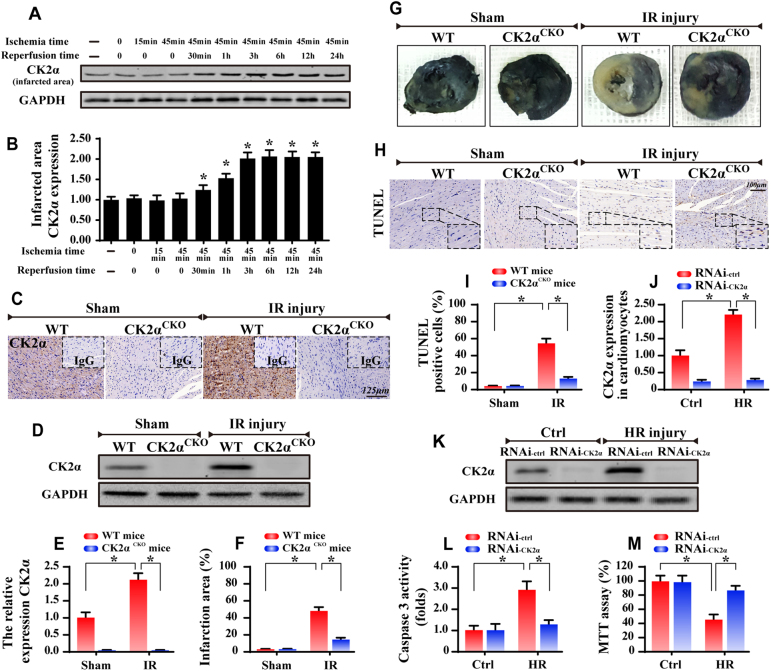
Upregulation of CK2α at the infarcted area. WT mice and CK2α^*CKO*^ mice underwent the 45-min ischemia and 0–24-hour reperfusion (IR injury, *n* = 6/group). In vitro, 45-min of hypoxia and 6-hour of reoxygenation (HR) was used to mimic the IR injury. Meanwhile, the loss-of-function assay about CK2α was conducted via siRNA (RNAi-CK2α) or control siRNA (RNAi-ctrl) in cardiomyocytes. **a** CK2α expression in the infarcted area. **b** Quantitative analysis of the relative expression of CK2α. **C**. The immunohistochemistry of CK2α in heart. **d**, **e** The western blots were used to confirm the CK2α expression in WT mice or CK2α^*CKO*^ mice under IR injury. **f**, **g** Representative images of heart sections with TTC and Evans Blue staining of the infarcted area. Bar graph indicates the infarct size. **h**, **i** TUNEL assay for cellular apoptosis analysis. **j**, **k** The control siRNA (RNAi-ctrl) and CK2α-siRNA (RNAi-CK2α) were transfected into cardiomyocytes. The transfection efficiency was confirmed by western blots. **l**, **m** Caspase3 activity and MTT assay were used to detect the cellular viability and apoptosis. The data represent the mean ± SEM. *P < 0.05

To observe the cardiac CK2α response to IR injury, mice were subjected to 45 min ischemia followed by 0–24 h of reperfusion. Our results suggested that CK2α transcription (Supplemental Fig. 1A) and expression (Fig. 1a, b) were progressively increased during the course of reperfusion. Notably, when compared to the normoxic and/or ischemic stages, 30 min of reperfusion significantly upregulated CK2α expression. Moreover, CK2α content was highest following 6 h of reperfusion. After that, prolonged duration of reperfusion failed to further upregulate CK2α expression (Fig. 1a, b). Similar results were obtained in cardiomyocytes in the setting of hypoxia and reoxygenation in vitro (Supplemental Fig. 1B-C). Therefore, reperfusion for 6 h was used in the remaining experiments. Subsequently, we asked whether CK2α had a causal role in regulating IR injury and thus cardiac-specific CK2α knockout (CK2α^*CKO*^) mice were employed. Western blots and immunohistochemistry were used to confirm the CK2α expression. IR upregulated the CK2α expression in WT mice but not in CK2α^*CKO*^ mice (Fig. 1c–e). Furthermore, CK2α^*CKO*^ mice demonstrated a significant reduction in infarction area compared with that in the WT group (Fig. 1f, g). TUNEL assay displayed that CK2α^*CKO*^ mice had fewer TUNEL-positive cells than mice in the WT group (Fig. 1h, i).

More solid evidence was obtained in primary cardiomyocytes with 45 min hypoxia followed by 6 h of reoxygenation (HR). CK2α expression was greatly increased after HR (Fig. 1j, k). Subsequently, the loss-of function assay for CK2α was carried out using siRNA, and knockdown efficiency was confirmed by western blots (Fig. 1j, k). Silencing of CK2α displayed no effect on cellular viability although it attenuated HR-induced cellular death as evidenced by casapse3 activity and MTT assay (Fig. 1l, m). To exclude the influence of siRNA transfection on HR-mediated cellular damage, cardiomyocytes were also isolated from WT and CK2α^*CKO*^ mice and were subjected to HR injury in vitro. Through TUENL assay (Supplemental Fig. 1D-E), HR was found to obviously increase TUNEL-positive cardiomyocytes isolated from WT mice; the effect of which was abolished in cells obtained from CK2α^*CKO*^ mice. These data indicate that upregulated CK2α likely participated in the IR injury via mediating cellular death.

### CK2α deficiency maintains cardiac function

**Fig. 2:**
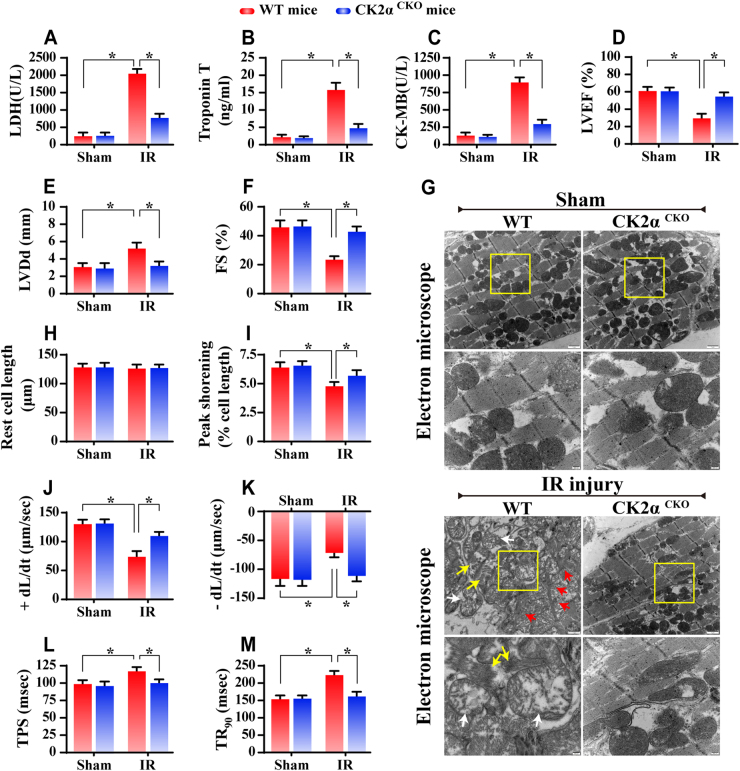
Loss of CK2α improved cardiac function. **a**–**c** The content of LDH, Troponin T, and CK-MB, which are markers of cardiac damage. **d**–**f** Cardiac function is evaluated through echocardiography. Quantitative analysis of the data derived from echocardiography. **g** EM was used to observe the ultrastructural changes after IR injury. Compared to the WT mice, the myofibrils in the CK2α^*CKO*^ mice were regularly arranged, and the Z lines were straight. Most mitochondria had normal structures. **h**–**m** The cardiomyocytes contractile properties in WT and CK2α^*CKO*^ mice in the context of IR injury *n* = 70–80 cells from 2 mice per group and experiments were repeated three times. The data represent the mean ± SEM. *P < 0.05. +dL/dt is the maximal velocity of shortening, −dL/dt maximal velocity of relengthening, FS fractional shortening, LVEF left ventricular ejection fraction, LVDd left ventricular diastolic dimension, TPS time-to-peak shortening, TR_90_ time-to-90% relengthening

Outside of cellular death, we further evaluated the changes of cardiac function. Compared with WT mice, the CK2α^*CKO*^ mice displayed less amounts of LDH, Troponin T and CK-MB (Fig. 2a–c). Meanwhile, the IR-suppressed cardiac function including left ventricular ejection fraction (LVEF) and left ventricular fractional shorting (LVFS) was restored to ~92 and ~95%, respectively, of the baseline levels, in CK2α^*CKO*^ mice (Fig. 2d–f). Moreover, left ventricular diastolic dimension (LVDd) was also significantly decreased in CK2α^*CKO*^ mice after IR injury when compared to that in IR-challenged WT mice (Fig. 2d–f).

To discern if functional improvement is originated from the structural protection, electron microscope (EM) was used to assess the architecture of myocardium (Fig. 2g). Our results indicate that IR injury triggered more mitochondrial damage (white arrows), myocyte dissolution (yellow arrows), muscular fiber twisting and Z line disappearance (red arrows); and these changes mostly disappeared in CK2α^*CKO*^ mice. Subsequently, cardiomyocyte properties were detected through acute isolation of cardiomyocytes from WT and CK2α^*CKO*^ mice after IR injury based on our previous study [25]. Neither IR injury nor CK2α deletion overtly affected the resting cell length in cardiomyocytes (Fig. 2h). However, cardiomyocytes from WT mice exposed to IR injury displayed significantly depressed PS and ±dL/dt as well as prolonged TPS and TR_90_ (Fig. 2i–m). In contrast, CK2α deficiency did not affect these mechanical parameters tested; it apparently reduced or abrogated IR-induced mechanical changes. To exclude the influence of fresh isolation on cellular function, spontaneous contraction was monitored in primary cardiomyocytes isolated from WT mice with or without CK2α siRNA silencing following HR treatment. Videos (Supplemental videos A-D) of cardiomyocytes beating display that loss of CK2α sustained the rhythm and amplitude cardiomyocyte contraction after HR injury. These data indicate that CK2α likely promoted cardiac dysfunction in IR injury.

### CK2α deficiency affords cardiomyocyte protection against mitochondria-related cell death

**Fig. 3:**
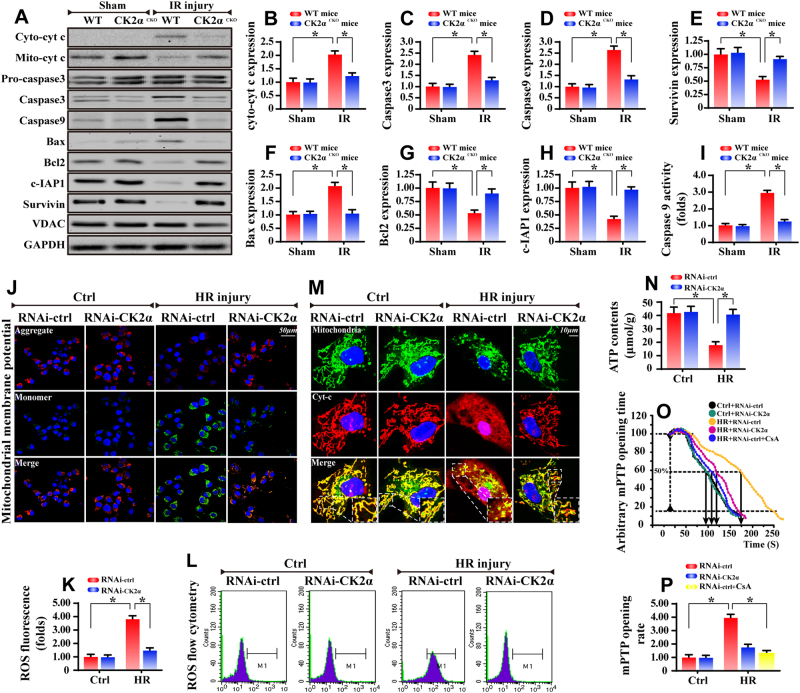
CK2α was implicated in cardiac mitochondrial apoptosis. **a**–**h** Western blots were used to analyze the proteins alterations related to the mitochondrial apoptosis in vivo. VDAC is the loading control of mitochondrial proteins. **i** The caspase 9 activity detection via ELISA assay. **j** In vitro, JC-1 staining was used to display the collapse of mitochondrial potential. **k**, **l** Cellular ROS content was quantified via flow cytometry analysis. **m** Immunofluorescence of cyt-c. Mitochondria were marked by Tom20. Nuclei were labeled by DAPI. **n** ATP production was analyzed via commercial kit. **o**, **p** mPTP opening time was determined as the time when the TMRE fluorescence intensity decreased by half between the initial and residual fluorescence intensity. CsA (Cyclosporine A) was used as the negative control group. The data represent the mean ± SEM. *P < 0.05

Mitochondrial death in cardiomyocytes is deemed the primary factor for the development of IR injury [26]. As shown in Fig. 3a–i, IR injury upregulates the expression of mitochondrial pro-apoptotic proteins and promotes caspase 9 activity, the effects of which were abrogated by CK2α deletion. Similar results were also obtained in primary cardiomyocytes with CK2α siRNA silencing in the setting of HR injury (Supplemental Fig. 2A-H), or in cardiomyocytes isolated from WT and CK2α^*CKO*^ mice subject to HR insult (Supplemental Fig. 2I).

Mitochondrial apoptosis is characterized by ΔΨm dissipation, cyt-c leakage, mPTP opening, ROS overproduction and ATP undersupply [27]. In vitro, HR treatment reduced the ΔΨm (Fig. 3j), which was accompanied with more ROS production via flow cytometry analysis (Fig. 3k, l). HR attack also caused more cyt-c leakage from the mitochondria into the cytoplasm, even into the nucleus, as revealed by immunofluorescence (Fig. 3m). In addition, HR suppressed ATP production (Fig. 3n) and increased the mPTP opening (Fig. 3o, p) in vitro. In contrast, with the loss of CK2α, HR-induced changes in ΔΨm reduction, ROS overproduction, cyt-c leakage, mPTP opening and ATP shortage were dramatically recused. Mitochondrial energy is vital for cellular survival in response to reperfusion injury. HR reduced the state 3/4 respiratory rates, ADP phosphorylation (respiratory control ratio), efficiency of ATP synthesis (ADP/O) and ADP phosphorylation lag phase (time elapsed in the depolarization/repolarization cycle during ADP phosphorylation) (Supplemental Fig. 2J-N). However, loss of CK2α reversed the mitochondrial respiratory function. These data substantiated that mitochondria are likely the target of CK2α under IR injury.

### FUNDC1-required mitophagy is upregulated in response to CK2α deletion

**Fig. 4:**
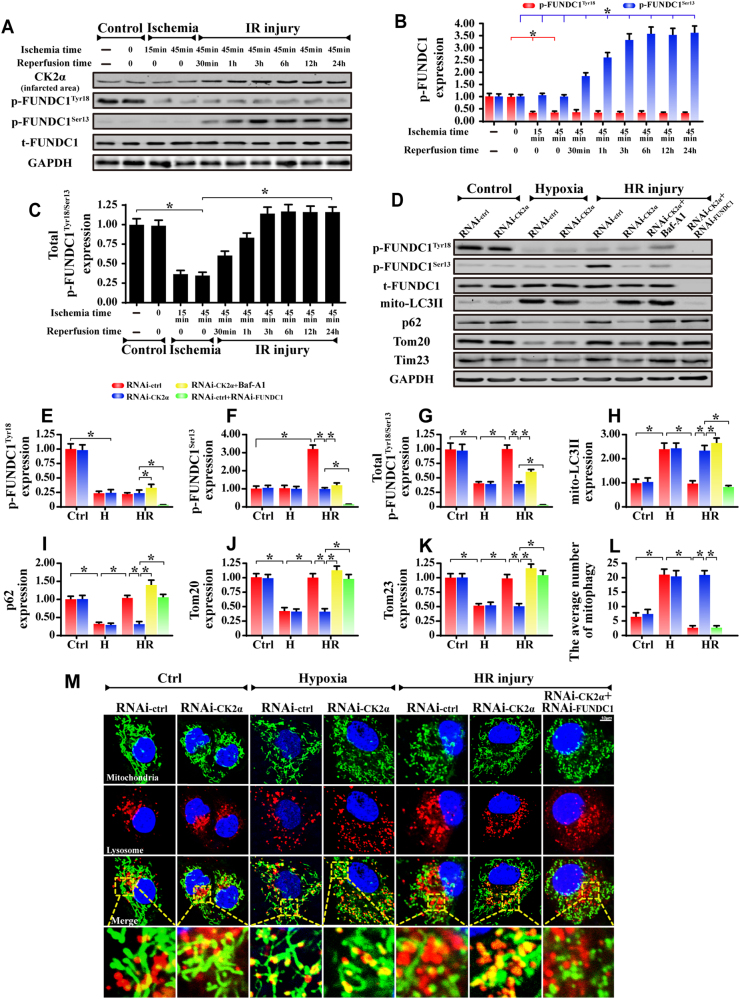
CK2α influenced FUNDC1-required mitophagy. **a**–**c** Proteins were isolated from heart tissues under normoxia (control group), ischemia and reperfusion conditions. The FUNDC1 phosphorylation was detected. **d**–**k** Proteins were isolated from cardiomyocytes with CK2α silenced in the presence of normoxia (control group), hypoxia and HR. FUNDC1 phosphorylation and mitophagy markers were analyzed via western blots. siRNA against FUNDC1 was used to establish the loss of function assay examining the role of FUNDC1 in CK2α-deleted cells. Bafilomycin-A1 (Baf-A1), the inhibitor of autophagolysosome, was used to evaluate the autophagy flux. **l**, **m** To directly observe changes in mitophagy, the co-immunofluorescence of mitochondria and lysosomes was used. Organ immunofluorescence was the hallmark of the interaction of mitochondria and lysosomes and was indicative of mitophagy. The data represent the mean ± SEM. *P < 0.05

FUNDC1-mediated mitophagy is beneficial for cardiac IR injury. However, phosphorylated FUNDC1 exerts steric hindrance for LC3II binding [18], thus strongly inhibiting mitophagy. As shown in Fig. 4a, b, at normoxia condition (control group), abundant p-FUNDC1^Tyr18^ were predominately found in cardiomyocytes whereas subtle p-FUNDC1^Ser13^ expression was observed, indicating that baseline FUNDC1 activity was primarily governed by p-FUNDC1^Tyr18^. In response to the ischemia, the level of p-FUNDC1^Tyr18^ was dramatically downregulated whereas p-FUNDC1^Ser13^ remained relatively unchanged, indicative of FUNDC1 activation by ischemia via p-FUNDC1^Tyr18^ downregulation. Furthermore, upon reperfusion exposure, ischemia-inhibited p-FUNDC1^Tyr18^ remained relatively unchanged whereas p-FUNDC1^Ser13^ was gradually upregulated, indicative of FUNDC1 inactivation by reperfusion via p-FUNDC1^Ser13^ upregulation. Through analysis of the levels of phosphorylated FUNDC1^Tyr18/Ser13^ (Fig. 4a, c), we demonstrated that the inactivated forms of FUNDC1 (total levels of p-FUNDC1^Tyr18/Ser13^), were firstly downregulated during ischemia but then were progressively upregulated in reperfusion. Given that no alterations were noted for p-FUNDC1^Tyr18^ at both ischemia and reperfusion stages, the increase in total levels of p-FUNDC1^Tyr18/Ser13^ in reperfusion phase is primarily attributable to enhanced p-FUNDC1^Ser13^. Similar results were also found in cardiomyocytes in vitro (Supplemental Fig. 3A-D).

The synchronous changes of CK2α and p-FUNDC1^Ser13^ in reperfusion, in conjunction with the fact that p-FUNDC1^Ser13^ is the substrate of CK2α, implies that reperfusion-upregulated CK2α may interrupt mitophagy through enhanced p-FUNDC1^Ser13^. Through loss-of-function assay of CK2α, we demonstrate that p-FUNDC1^Tyr18^ level is only controlled by hypoxia. CK2α silence had no effects on its content regardless of normoxia, hypoxia and reoxygenation (Fig. 4d–g). In comparison, little p-FUNDC1^Ser13^ level was identified under normoxia and hypoxia conditions. Its expression was mainly increased by reoxygenation; this effect was reversed by CK2α knockdown (Fig. 4d–g). These data illustrate that baseline CK2α failed to induce FUNDC1 phosphorylation (inactivation) whereas reperfusion-upregulated CK2α exclusively promoted FUNDC1 phosphorylation at Ser13.

Furthermore, mitophagy activity is also elevated under hypoxia as evidenced by higher mito-LC3II, less p62 and lower Tim23 (mitochondrial inner membrane marker) and Tom20 (outer membrane marker) (Fig. 4d, h–k). In contrast, reoxygenation unfortunately repressed mitophagy, the effect of which was nullified by CK2α deletion (Fig. 4d, h–k). Through analysis of the mitophagic flux using bafilomycin-A1 (Baf-A1), we re-confirmed that CK2α deletion was able to augment mitophagy activity (Fig. 4d–k). Interestingly, under loss of FUNDC1, the beneficial role of CK2α deficiency in mitophagy activation disappeared (Fig. 4d–k). Similar results were also obtained in WT and CK2α^*CKO*^ mice (Supplemental Fig. 4A-F).

To directly assess mitophagy activity, immunofluorescence assay of mitochondria and lysosome was used (Fig. 4l, m). Most fragmented mitochondria cannot be engulfed by lysosomes following HR treatment, as demonstrated by sporadic co-localization of mitochondria and lysosomes, indicating mitophagy inhibition. These changes were reversed by CK2α deletion, which contributed to the fusion of mitochondria and lysosomes. Furthermore, once FUNDC1 expression was lost, the overlap of lysosome and mitochondria was re-inhibited in CK2α-deleted cells. Similar results were also observed in cardiomyocytes isolated from WT and CK2α^*CKO*^ mice (Supplemental Fig. 4G-H).

### **Genetic inhibition of FUNDC1 abolishes the protection observed in CK2α**^***CKO***^**mice**

**Fig. 5:**
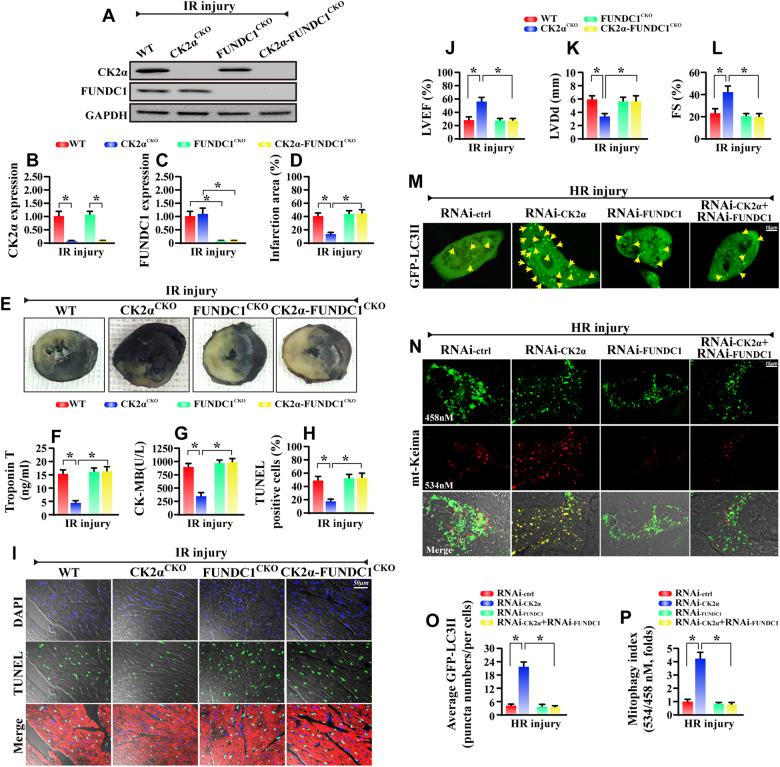
Deletion of cardiac FUNDC1 abrogated the cardio-protection observed in CK2α^*CKO*^ mice under IR. **a**–**c** Western blots was used to confirm the proteins changes in CK2α^*CKO*^ mice, FUNDC1^*CKO*^ mice and CK2α-FUNDC1^*CKO*^ mice. **d**, **e** The infarction area was measured after 45-min ischemia and 6-hour reperfusion. **f**, **g** The cardiac damage markers were detected via ELISA assay. **h**, **i** The cellular apoptosis was evaluated via TUNEL assay. The red fluorescence is the staining of Troponin T. **j**–**l** The cardiac function was detected via echocardiography. **m**, **o** GFP-LC3 transfection was used to detect mitophagy activity. The LC3II puncta were counted. **n**, **p** The mt-Keima assay was used to detect the acid mitochondria which is the result of fusion of mitochondria and lysosome. The orange mitochondria were counted. The data represent the mean ± SEM. *P < 0.05

To further confirm whether FUNDC1 activity is required for the protective effect of CK2α depletion in vivo, CK2α-FUNDC1 double-deficient mice were generated. Expected reduction in CK2α and FUNDC1 proteins were confirmed using western blot (Fig. 5a–c), and then these mice were subjected to IR injury. Consistent with our findings above, the CK2α^*CKO*^ mice had reduced infarcted area (Fig. 5d, e), improved cardiac function (Fig. 5f, g, j–l) and less TUNEL^+^ cells (Fig. 5h, i) when compared to the WT mice under IR injury. Interestingly, the CK2α-FUNDC1 double-deficient mice displayed increased infarcted zone, impaired cardiac function and more abundant cell death compared with CK2α^*CKO*^ mice (Fig. 5d–l). As depicted in our EM ultrastructural evaluation, genetic ablation of FUNDC1 re-induced mitochondrial vacuolization and myocyte dissolution when compared to the CK2α^*CKO*^ mice (Supplemental Fig. 5A).

Similarly, in vitro, we silenced FUNDC1 in CK2α-deleted cardiomyocytes, and the results indicate that the loss of FUNDC1 in CK2α-deleted cardiomyocytes caused more cell death via mitochondrial damage, as evidenced by more TUNEL^+^ cells (Supplemental Fig. 5B-C), increased caspase3 activity (Supplemental Fig. 5D) and more mitochondrial pro-apoptotic proteins (Supplemental Fig. 5E-I).

As for mitophagy, through the GFP-LC3 transfection, we demonstrate that less LC3II accumulates in HR-treated cells, and this change were recused by CK2α deletion (Fig. 5m, o). In contrast, with the knockdown of FUNDC1 in CK2α-deficiency cells, the number of punctate LC3II is reduced again (Fig. 5m, o). To provide more evidence for mitophagy activity, mt-Keima assay was used. More acid mitochondria were found in CK2α-deleted cells compared to the HR group. However, loss of FUNDC1 abated the number of acid mitochondria despite deletion of CK2α (Fig. 5n, p). These data demonstrate that FUNDC1 was necessary for the beneficial action of CK2α deletion via mitophagy.

### FUNDC1-required mitophagy protects mitochondrial structure and function

**Fig. 6:**
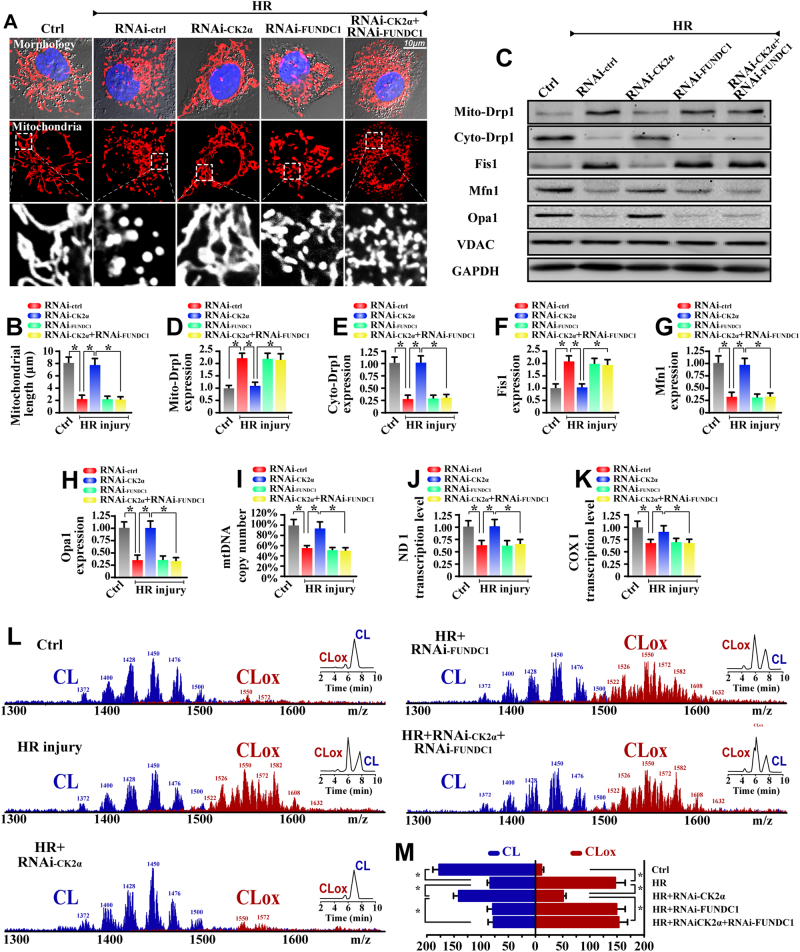
FUNDC1-required mitophagy protected the mitochondrial homeostasis under IR injury. **a** Cardiomyocytes were labeled with Tom20 to determine mitochondrial fragmentation. **b** To assess changes in mitochondrial morphology quantitatively, the length of mitochondria was measured. **c**–**h** Proteins related to mitochondrial fission and fusion were evaluated via western blots. **i** mtDNA copy number was assessed by complex IV segment. **j**, **k** The transcript level of mtDNA was reflected by two different components: NADH dehydrogenase subunit 1 (ND1) and cytochrome c oxidase subunit I (COX I). **l**, **m** Assessment of molecular species of CL and its oxidation products. The left panel indicates the non-oxidized (blue) and the appearance of numerous oxidized (red) CL species after HR attack. Right insets: 2-dimensional chromatographic separation of non-oxidized and oxidized CL (CLox). The data represent the mean ± SEM. *P < 0.05

To explore the possible protective role of FUNDC1 in IR injury, mitochondrial fragmentation was monitored based on the above observation that more round mitochondrial debris appeared in HR-treated cells. As shown in Fig. 6a, b, after treatment with HR, mitochondria divided into several fragments and that this configuration change was reversed by CK2α deletion. Furthermore, loss of FUNDC1 would re-induce mitochondrial debris in CK2α-deleted cells. Furthermore, the proteins related to mitochondrial fission including dynamin-related protein 1 (Drp1) and mitochondrial fission protein 1 (Fis1) were increased in HR treatment but reduced in CK2α-deleted cells (Fig. 6c–h). However, loss of FUNDC1 re-instated the accumulation of mito-Drp1 and Fis1 despite deletion of CK2α. On the other hand, the mitochondrial fusion proteins such as mitofusin 1 (Mfn1) and optic atrophy 1 (Opa1) were reduced in HR treatment but reversed to normal levels in CK2α-deleted cells in a FUNDC1-dependent manner (Fig. 6c–h). Similar results were also found in vivo experiments (Supplemental Fig. 6). These data indicate that loss of CK2α inhibits fission and promotes fusion via FUNDC1-required mitophagy which powerfully cleared mitochondrial fragmentations.

The key feature of mitochondrial homeostasis is mitochondrial genome stability. However, the mtDNA copy number (Fig. 6i) and mtDNA transcripts (Fig. 6j–k) were downregulated in HR-treated cardiomyocytes, and these changes were reversed by CK2α deletion. However, upon knockdown of FUNDC1 in CK2α-deleted cells, the mtDNA copy and transcription were reduced again. The electron transport chain complexes’ (ETC) activity is mainly handled by mtDNA. However, HR reduced the activity of ETC (Supplemental Fig. 7A-C), which was reversed via CK2α deletion, whereas inhibition of FUNDC1 canceled the protective action of CK2α deletion.

As for mitochondrial damage, we focused on cardiolipin (CL) oxidation which occurs at the early stage of mitochondrial apoptosis via liberation of cyt-c into cytoplasm. Using 2-dimensional high-performance thin-layer chromatography, we performed a global lipidomic analysis of CL (Fig. 6l, m), which demonstrated that of ≈185 individual molecular species of CL in normal cardiomyocytes, only ≈10 were oxygenated. Notably, HR induced the oxidation of the majority of the polyunsaturated molecular species of CL; the number of non-oxidized CL species decreased to ≈94, whereas the number of oxygenated species increased to ≈154 (Fig. 6l, m). Loss of CK2α reversed the ratio of non-oxidized CL, and these effects were nullified by FUNDC1 knockdown. Similar results were obtained via mitochondrial potential staining (Supplemental Fig. 7D-E), ROS oxidative stress detection (Supplemental Fig. 7F-G), mPTP opening assay (Supplemental Fig. 7H), and mitochondrial apoptotic proteins measurement (Supplemental Fig. 7I-M). These data indicate that loss of FUNDC1-related mitophagy aggravates mitochondrial damage.

Considering that the mitochondrial mass was reduced whereas the mtDNA copy/transcription was increased, which was accompanied with the decline in oxidized CL, we guessed whether FUNDC1-required mitophagy promoted the mitochondrial biogenesis. As shown in Supplemental Fig. 7N-P, peroxisome proliferator-activated receptor gamma coactivator 1-alpha (PGC1a), nuclear respiratory factor 1 (NRF1), and mitochondrial transcription factor A (TFAM) transcriptions were reduced in HR-treated cells but increased in CK2α-deleted cells. Interestingly, FUNDC1 deficiency re-evoked a deep decline in PGC1a, NRF1, and TFAM transcriptions, suggesting that FUNDC1-related mitophagy promoted the mitochondrial turnover via upregulation of mitochondrial biogenesis.

## Discussion

In this study, we uncover that (1) CK2α is progressively increased in the reperfused heart, which is instrumental for scar expansion and cardiac dysfunction in response to IR injury; (2) functional assays illustrate that higher CK2α expression renders cardiomyocytes to mitochondrial apoptosis via post-transcriptional inactivation of FUNDC1, leading to the inhibition of protective mitophagy; (3) defective mitophagy fails to remove the damaged mitochondria induced by IR injury, resulting in mitochondrial genome collapse, ETC inactivity, cardiolipin oxidation and mitochondrial apoptosis; (4) loss of cardiac CK2α sustains heart function and structure under IR injury, however, (5) genetic ablation of FUNDC1 abolishes the protection observed in CK2α deletion in vivo and in vivo. Collectively, IR promoted disturbance in mitochondrial integrity, thus demanding well-orchestrated mitophagy to remove damaged mitochondria. Unfortunately, FUNDC1-required mitophagy was suppressed via reperfusion by way of CK2α, leading to the progression of IR injury. As far as we know, this is the first study to identify CK2α-FUNDC1 mitophagy pathways as the pathogenesis of cardiac IR injury via governing mitochondrial homeostasis.

Mitochondrial damage is conclusively involved in cardiac IR injury via multiple mechanisms [26]. The key finding in the present study is that CK2α is a novel molecular basis for disturbed mitochondrial homeostasis in IR injury. Protein kinase CK2α participates in heart and neural tube development, maintains cell viability, and regulates cell cycle stages [28, 29]. Many studies have demonstrated that CK2α regulates the mitochondrial oxidative [30] and induces p53-related mitochondrial apoptosis [31]. Although previous studies have confirmed the harmful effects of CK2α on chronic cardiac injury including diabetic cardiomyopathy [23] and heart failure [24], its detailed molecular machinery related to mitochondrial etiology in cardiac IR injury remains largely unexplored. In the present study, we demonstrate that CK2α is activated by IR injury contributing to FUNDC1 inactivation and mitophagy arrest. This finding introduces a role for CK2α-mediated mitophagy in acute cardiac damage for the first time, which should help to fill the gap in the current knowledge regarding the molecular links between CK2α, mitophagy, mitochondrial dysfunction and cardiac IR injury.

The harmful insults of CK2α on mitochondria depends on FUNDC1-related mitophagy activity. First, reperfusion injury definitely induced mitochondrial damage via promoting mitochondrial fission [32], impairing the mtDNA genome stability [33], reducing ETC activity, evoking mitochondrial oxidative stress [34], and more severely, initiating mitochondrial apoptosis [35, 36]. Despite damage to mitochondria, cells could utilize well-orchestrated mitophagy to timely remove the bad mitochondria and largely sustain the mitochondrial respiratory function [37]. Although FUNDC1-required mitophagy has been reported to be activated by ischemia/hypoxia [16, 22, 38], its activity was unfortunately downregulated in reperfusion through poorly defined mechanisms [3, 39]. Our findings propose that FUNDC1-required mitophagy was activated by ischemia via p-FUNDC1^Tyr18^ downregulation but inhibited by reperfusion through upregulated CK2α and its substrate p-FUNDC1^Ser13^, redefining the paradigm of FUNDC1/mitophagy activation and inactivation during ischemia and reperfusion, respectively. Thereby, this information clearly explains the molecular mechanism responsible for the defective FUNDC1-required mitophagy in the context of reperfusion injury.

The process of mitophagy activation relies on a growing cadre of “mitophagy adaptors”, and three regulators have been reported according to our previous findings: FUNDC1 [3, 7], Bnip3 [6, 8, 9] and Parkin [14]. More importantly, different receptors may have various influences on cell fate, ranging from cellular protection to cellular self-destruction. In answer to cardiac IR injury, excessive Parkin- and Bnip3-involved mitophagy exacerbated cardiomyocytes death [9, 14] whereas FUNDC1-related mitophagy provides beneficial effects on cell survival [3, 7]. A recent study [40] has demonstrated that CK2β deletion was associated with mitochondrial dysfunction and activated Parkin-mediated mitophagy due to the impairment of PINK1 importing into mitochondria and blocking autophagic flux. Their finding is similar to our previous study that excessive Parkin-induced mitophagy augmented mitochondrial injury. Accordingly, the discrepancy existing with regard to the detailed role of mitophagy in mitochondrial homeostasis is principally attributed to the various mitophagy adaptors.

At molecular level, FUNDC1 signals mitophagy to sweep the mitochondrial debris, sustain mitochondrial respiratory and block mitochondrial apoptosis. More importantly, although FUNDC1-required mitophagy promotes the clearance of mitochondrial mass, the mtDNA copy/transcription is actually increased, which was accompanied with the elevated mitochondrial biogenesis, leading to the mitochondrial turnover or renewing. These results correlate well with previous conclusion that mitophagy is required for mitochondrial biogenesis via accelerating mitochondria turnover [41, 42]. However, the exact mechanism by which mitophagy promotes the mitochondrial biogenesis remains unclear.

In conclusion, we answer the question of how CK2α aggravates the development of IR injury: CK2α cuts off FUNDC1-reqiured mitophagy and impairs the mitochondrial protective system, eventually amplifying the cardiomyocytes death signals.

## Methods

### Animal models of cardiac IR Injury

All animal procedures were performed in accordance with the Guide for the Care and Use of Laboratory Animals, which was published by the US National Institutes of Health (NIH Publication No. 85-23, revised 1996) and were approved by the University of Wyoming Institutional Animal Use and Care Committee (Laramie, WY, USA). We first generated CK2α^*fl/fl*^ mice as previously reported [43]. The detailed information is described in the supplemental methods. The generation of FUNDC1^*fl/fl*^ mice was as our previous study described. The cardiac-specific FUNDC1 knockout (FUNDC1^*CKO*^) and CK2α knockout (CK2α^*CKO*^) mice were generated via CK2α^*fl/fl*^ or FUNDC1^*fl/fl*^ mice with α-MHC (alpha myosin heavy chain)-Cre transgenic mice. Subsequently, FUNDC1^*fl/fl*^ were bred to CK2α^*CKO*^ mice to generate cardiac-specific CK2α-FUNDC1 double knockout (CK2α-FUNDC1^*CKO*^, CK2α^*fl/fl*^; FUNDC1^*fl/fl*^; Cre^*α-MHC*^) mice. To avoid potential variation related to gender, all experiments were performed in male mice. These mice (8 weeks old) were used to induce an IR injury model (45 min ischemia and 0–24 h reperfusion) according to our previous studies [2]. Upon the completion of the reperfusion, the hearts were stained with 2% Evans Blue and 1% 2,3,5-triphenyltetrazolium chloride. The infarct size was expressed as a percentage of the risk zone (*n* = 6/group in one experiment). The content of lactate dehydrogenase (LDH), Troponin T and creatine kinase-MB (CK-MB) in the blood was evaluated via ELISA assays as our previous study described [2]. Details on echocardiogram, cell shortening/relengthening assay, electron microscopy and TUNEL assay are described in the supplemental methods.

### Cell culture, HR injury induction and siRNA assay

The HR injury model was mimicked in vitro by 45 min of hypoxia and 6 h of reoxygenation. The primary cardiomyocytes were isolated from WT and CK2α^*CKO*^ mice according to our previous study [44]. To inhibit the mitophagy, siRNA against FUNDC1 was transfected to cardiomyocytes isolated from WT mice. To observe the autophagic flux, Bafilomycin-A1 (0.5 μM, Selleck Chemicals) was used 12 h before treatment. Details on MTT assay, TUNEL staining and caspase3/9 activities are described in the supplemental methods. The siRNAs specific against the expression of CK2α and FUNDC1 or control siRNAs were purchased Santa Cruz Biotechnology. The siRNA transfection was based on our previous study [45], and the transfection efficiency was confirmed by western blots.

### Mitophagy detection

Mitophagy was observed via immunofluorescence by co-staining of mitochondria and lysosome. The mitophagy flux was measured by detecting GFP-LC3II puncta through transfection of recombinant adenovirus GFP-LC3 under laser scanning confocal microscopy (Nikon A1R, Japan). Mt-Keima is a ratiometric pH-sensitive fluorescent protein that is targeted into the mitochondrial matrix. A low-ratio mt-Keima derived fluorescence (543/458 nm) reports neutral environment, whereas a high-ratio fluorescence reports acidic pH. Thus, mt-Keima enables differential imaging of mitochondria in the cytoplasm and mitochondria in acidic lysosomes. Mitochondria-targeted mKeima-Red expression plasmid (pMT-mKeima-Red, #AM-V-251, MBL Medical & Biological Laboratories, Co., ltd. Woburn, MA) was transfected to cells. Ratio (543/458 nm) of mKeima emission light were calculated as a value of mitophagy. Details on ROS measurement, ATP production, and mitochondrial respiratory function are described in the supplemental methods.

### Western blotting and immunofluorescence

To detect the protein expression, immunoblotting was carried out. Briefly, samples were lysed in RIPA buffer containing 1 mM PMSF. After centrifugation, 30–60 μg protein for each sample was loaded and transferred to PVDF membrane. For immunofluorescence, samples were firstly washed with PBS, and then fixed with 4% paraformaldehyde for 30 min. Subsequently, samples were incubated with the primary antibody at 4 °C overnight. Then, samples were stained with fluorescent second antibody for 30 min. DAPI was used for nuclear staining. The pictures were acquired under confocal microscopy. The primary antibodies used in the present study are described in the supplemental methods.

### Cardiolipin extraction and high-performance thin-layer chromatography analysis

The mitochondria were used to obtain cardiolipin by high-performance thin-layer chromatography with an electrospray ionization source and a linear ion trap mass spectrometer (LXQ Thermo-Fisher). To avoid the oxidation of phospholipids during separation, chloroform/methanol (2/1, v/v) containing 0.05% BHT as antioxidant was added. The cardiolipin and its oxidized molecular species were extracted, separated and analyzed based on our report [2].

### The mtDNA copy/transcription, mPTP opening, and mitochondrial potential (ΔΨm) detection

The relative amounts of mitochondrial DNA (mtDNA) and nuclear DNA (nDNA) content were used to assess the mtDNA copy numbers via PCR [2]. The mtDNA and nuclear amplicons were generated from a complex IV segment and GAPDH segment, respectively. The transcript level of mtDNA was reflected by two different components: NADH dehydrogenase subunit 1 (ND1) and cytochrome c oxidase subunit I (COX I). The primers are described in the supplemental methods. Complex I, II, and IV activity was measured according to previous studies [46].

The opening of the mPTP was visualized as a rapid dissipation of tetramethylrhodamine ethyl ester fluorescence. Arbitrary mPTP opening time was determined as the time when tetramethylrhodamine ethyl ester fluorescence intensity decreased by half between initial and residual fluorescence intensity according to our previous study [2].

The ΔΨm was analyzed using JC-1 staining (Beyotime Institute of Biotechnology). Briefly, cells were washed with ice-cold PBS and then stained with 2.5 g/ml JC-1 for 30 min at 37 °C. After being washed with binding buffer, the cells were analyzed by fluorescence microscopy. Results are presented as relative aggregate-to-monomer (red/green) fluorescence intensity ratio.

### Statistical analysis

Data analysis was conducted using SPSS 19.0 statistical software (SPSS, Chicago, IL, USA). Measurement data were presented as mean ± SEM. The one-way analysis of variance was conducted among multi-groups. *P* < 0.05 indicated statistical significance.

## Electronic supplementary material

Supplemental Figures

Supplemental methods

Video A

Video B

Video C

Video D
