# Supplementary material for: Pathogenesis of cardiac ischemia reperfusion injury is associated with CK2α-disturbed mitochondrial homeostasis via suppression of FUNDC1-related mitophagy
Source: Cell Death Differ. 2018 Mar 14;25(6):1080–93. doi: 10.1038/s41418-018-0086-7 (PMC5988750; doi:10.1038/s41418-018-0086-7)

**Supplemental Figures for**

**Pathogenesis of cardiac ischemia reperfusion injury is associated with CK2α-disturbed mitochondrial homeostasis via suppression of FUNDC1-related mitophagy**

**Running title**: CK2α controls cardiac IR injury via mitophagy.

**Supplemental figure1** CK2α was increased in the reperfused heart. **A.** The mRNA transcription of CK2α. **B-C.** Cardiomyocytes were isolated from WT mice and underwent the HR injury. CK2α expression was detected via western blots. **D-E.** Cardiomyocytes were obtained from WT and CK2α*^CKO^* mice, and the TUNEL assay was used to analyze the cellular death. The data represent the mean±SEM. *P<0.05.

**
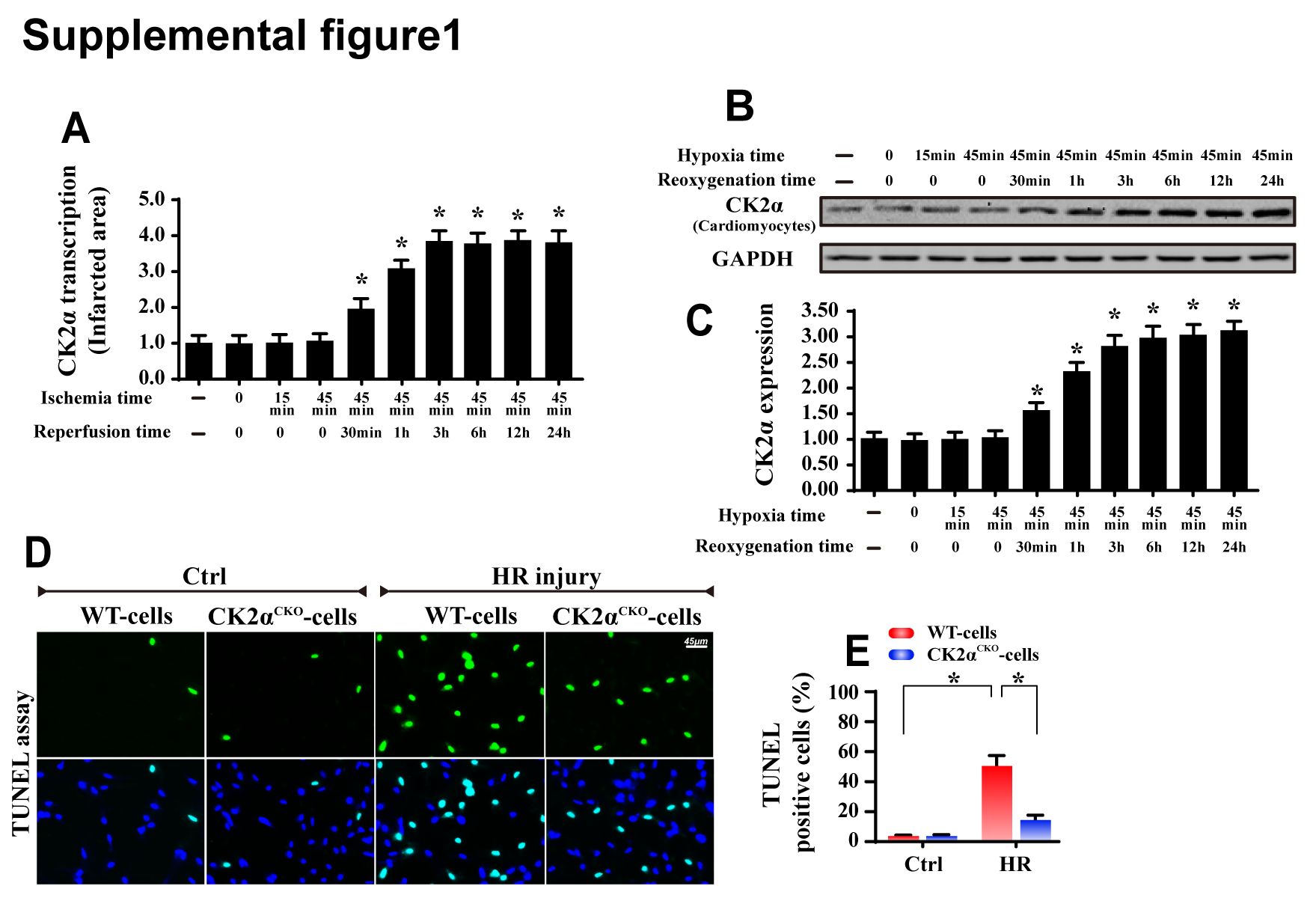
**

**Supplemental figure2** CK2α was implicated in mitochondrial apoptosis in vitro. **A-H.** Western blots were used to analyze the proteins alterations related to the mitochondrial apoptosis. VDAC is the loading control of mitochondrial proteins. **I.** Cardiomyocytes were obtained from WT and CK2α*^CKO^* mice, and the caspase9 activity was measured. **J-N.** Effect of CK2α on state 3 respiration, state 4 respiration, respiratory control ratio (RCR [state 3/state 4]), number of nmol ADP phosphorylated to atoms of oxygen consumed (ADP/O), and ADP phosphorylation lag phase (time elapsed in the depolarization/repolarization cycle during ADP phosphorylation). The data represent the mean±SEM. *P<0.05.

**
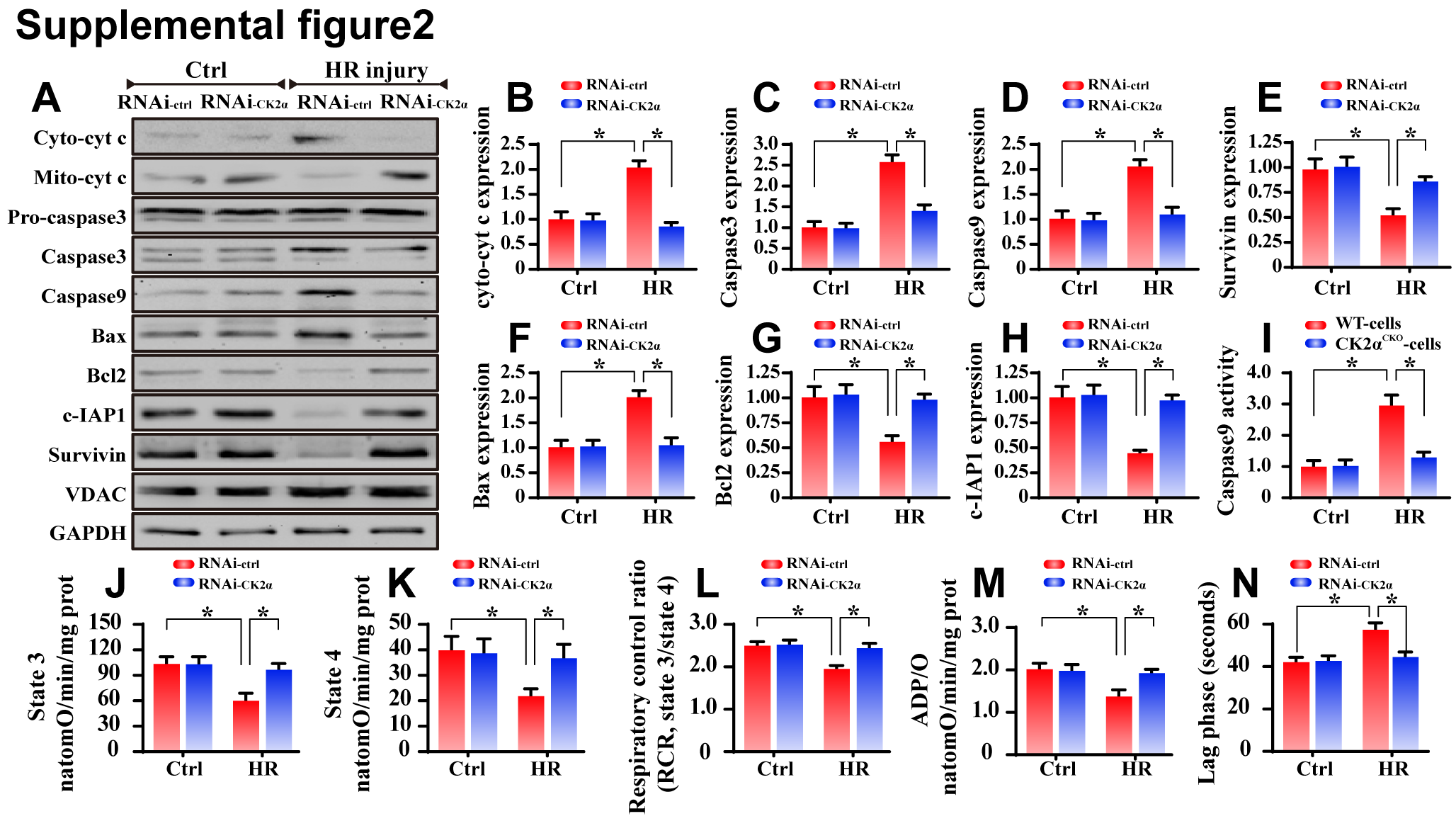
**

**Supplemental figure3** p-FUNDC1^Ser13^ was increased in response to the reoxygenation in vitro. **A-D.** Proteins were isolated from cardiomyocytes in the presence of normoxia (control group), hypoxia and HR. FUNDC1 phosphorylation was detected. The data represent the mean±SEM. *P<0.05.

**
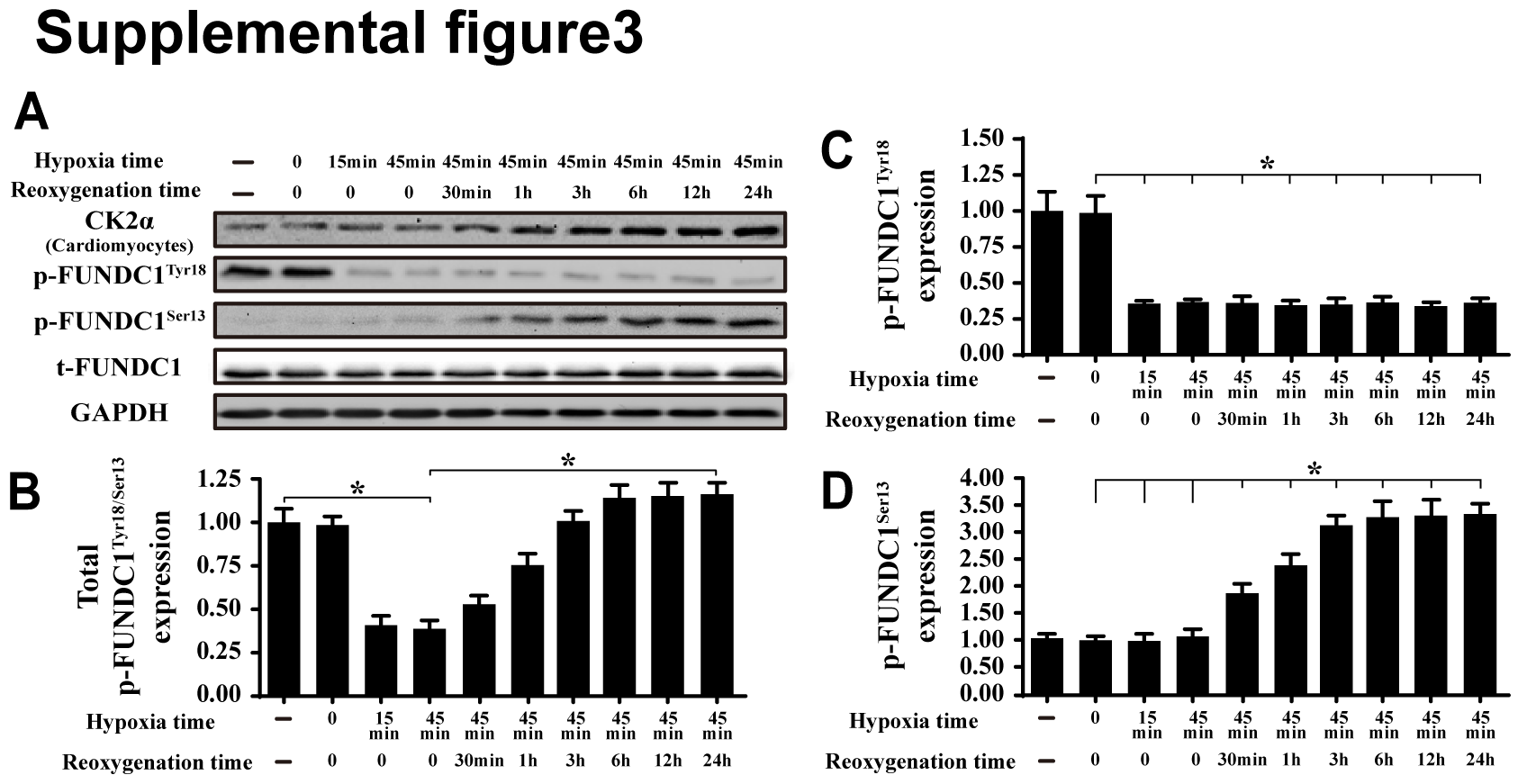
**

**Supplemental figure4** CK2α regulated FUNDC1-required mitophagy in vivo. **A-F.** Western blots of p-FUNDC1^Ser13^ and mitophagy parameters in myocardial samples from WT mice or CK2α*^CKO^* mice under IR injury. **G-H.** Cardiomyocytes were obtained from WT and CK2α*^CKO^* mice, and the mitophagy activity was measured via co-staining of mitochondria and lysosome. The data represent the mean±SEM. *P<0.05.

**
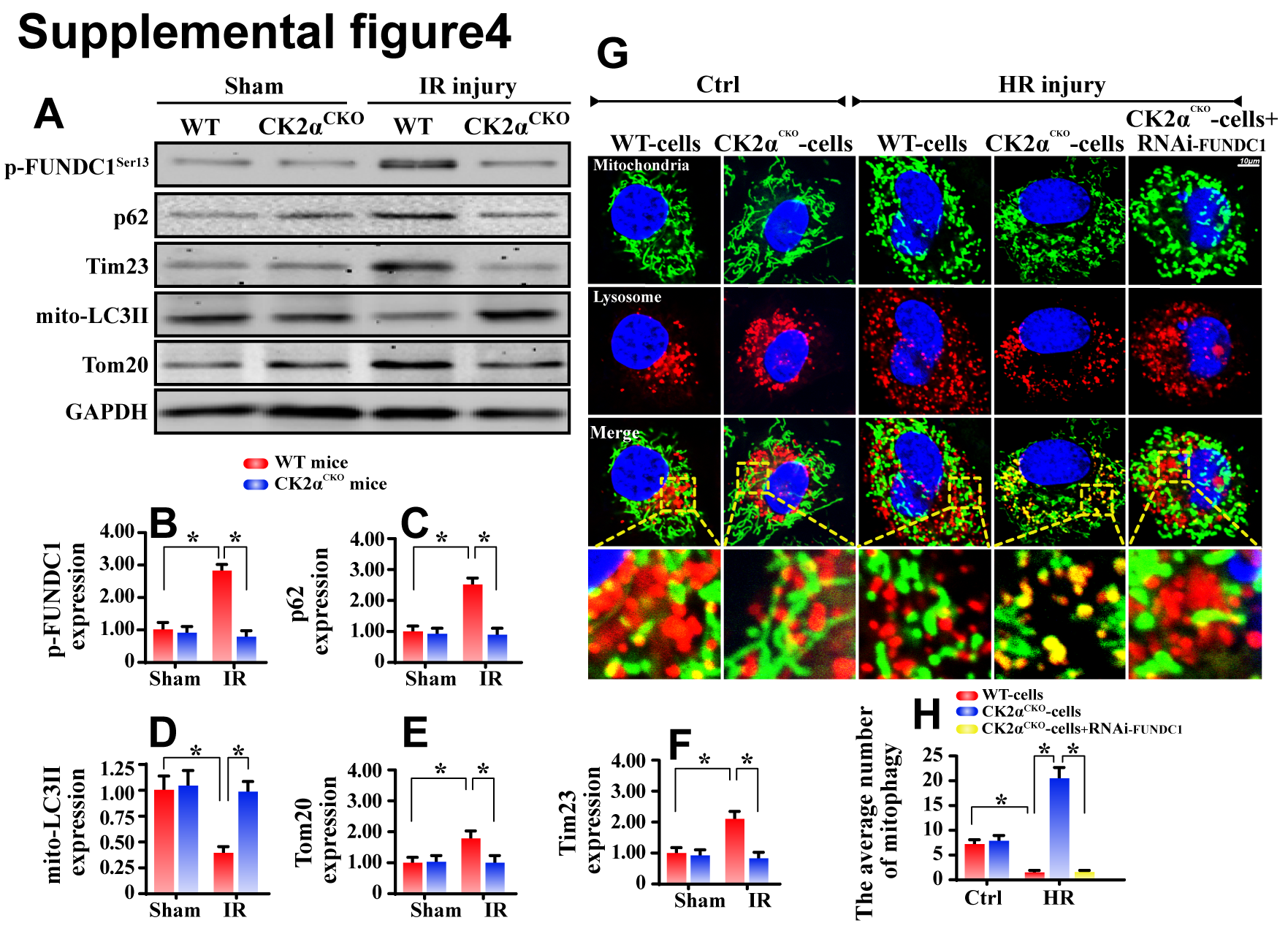
**

**Supplemental figure5** Loss of FUNDC1 attenuated the protective effect of CK2α silencing. **A.** EM was used to observe the change of cardiac structure and mitophagy. The yellow arrows indicate the damaged or vacuolated mitochondria. The red arrows indicate the mitophagy. **B-C.** TUNEL assay was used to detect the cellular apoptosis. **D.** The changes of casapse3 activity. **E-I.** The mitochondrial apoptotic proteins were detected via western blots. The data represent the mean±SEM. *P<0.05.

**
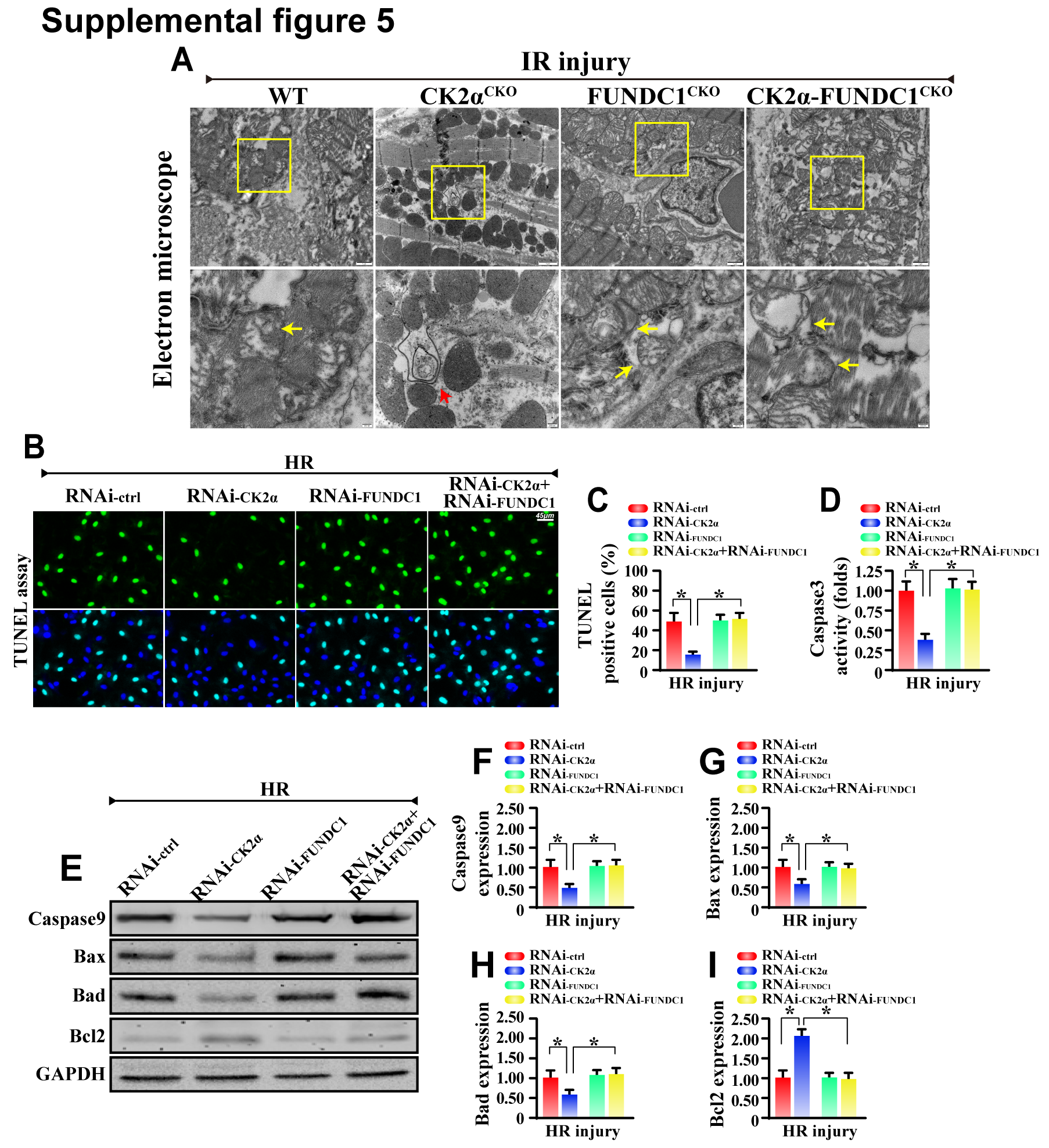
**

**Supplemental figure6** Deletion of cardiac FUNDC1 promoted the accumulation of mitochondrial fragmentations in vivo. **A-F.** Western blots was used to confirm the fission- and fusion-related proteins changes in CK2α*^CKO^* mice, FUNDC1*^CKO^* mice and CK2α-FUNDC1*^CKO^* mice. The data represent the mean±SEM. *P<0.05.

**
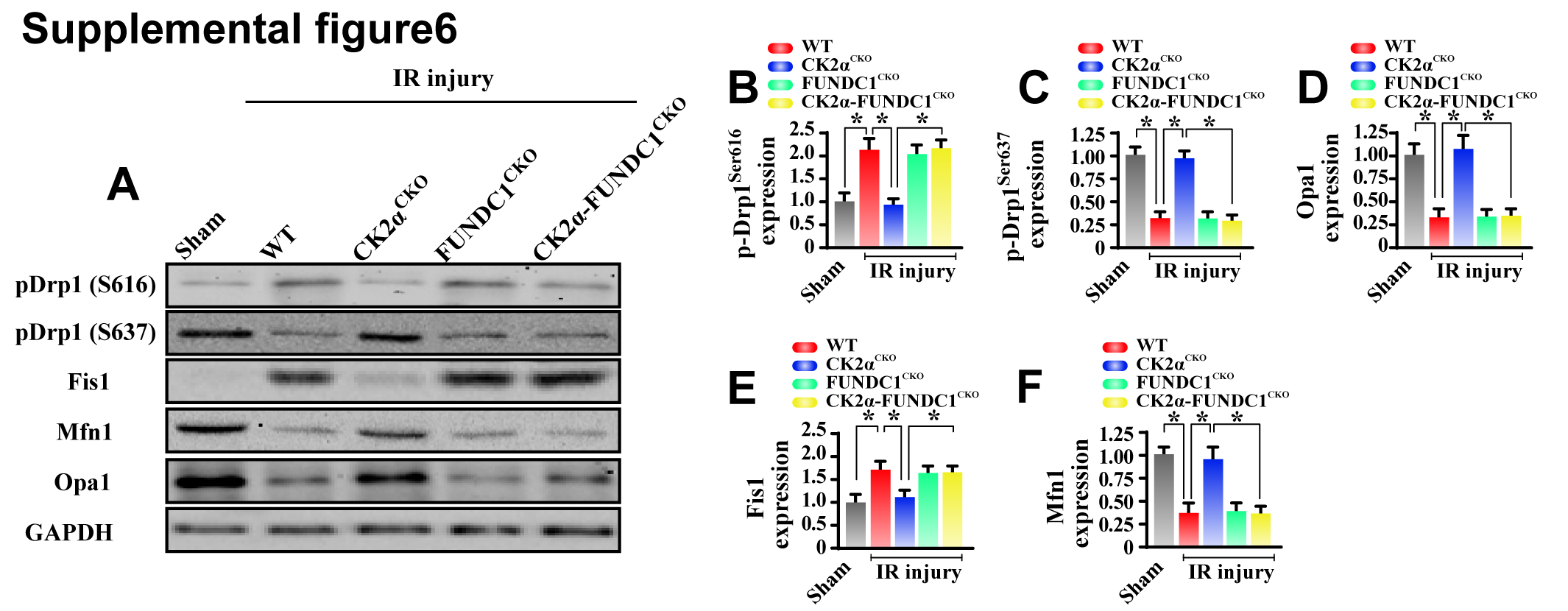
**

**Supplemental figure7** FUNDC1 maintained the mitochondrial homeostasis. **A-C.** The change of ETC activity was detected via commercial kit. **D-E.** The mitochondrial membrane potential was detected via JC1 staining. **F-G.** ROS production was detected via DCFH-DA staining. **H.** mPTP opening was measured. **I-M.** Mitochondrial apoptosis was detected via western blots. **N-P.** The changes of mitochondrial biogenesis markers. The data represent the mean±SEM. *P<0.05.


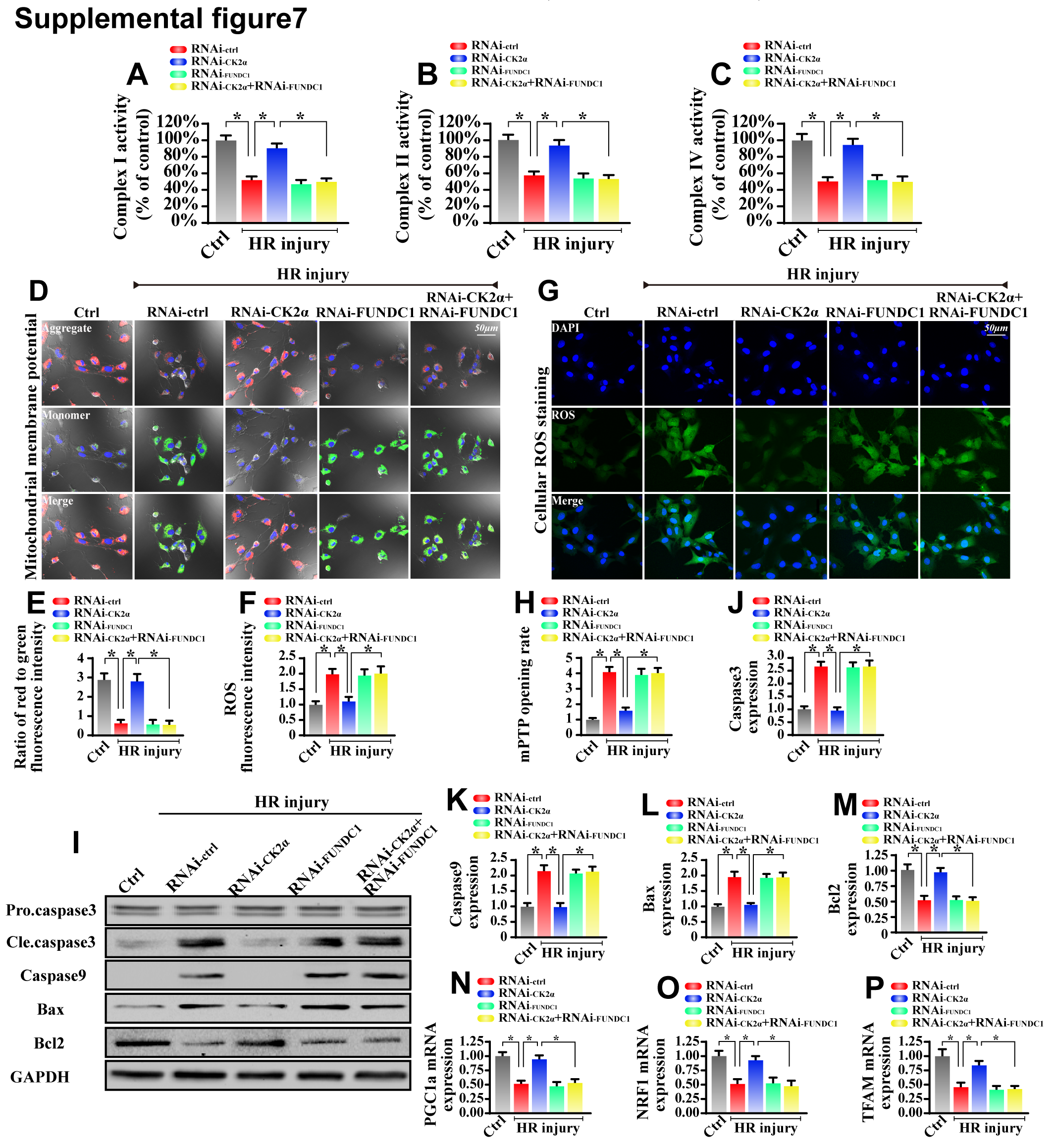

Supplement: Supplementary file 1 — Supplemental Figures [file 41418_2018_86_MOESM1_ESM.docx]
