# Supplementary material for: Pathogenesis of cardiac ischemia reperfusion injury is associated with CK2α-disturbed mitochondrial homeostasis via suppression of FUNDC1-related mitophagy
Source: Cell Death Differ. 2018 Mar 14;25(6):1080–93. doi: 10.1038/s41418-018-0086-7 (PMC5988750; doi:10.1038/s41418-018-0086-7)
Supplement: Supplementary file 2 — Supplemental methods [file 41418_2018_86_MOESM2_ESM.docx]

**Supplemental Methods for**

**Pathogenesis of cardiac ischemia reperfusion injury is associated with CK2α-disturbed mitochondrial homeostasis via suppression of FUNDC1-related mitophagy**

**Running title**: CK2α controls cardiac IR injury via mitophagy.

1. **Animal**

The floxed CK2α mice were generated according to the previous report ^1^. For CK2α, exon 2 was knocked out by homologous recombination. These exons were chosen because they contain the adenosine triphosphate binding site. Their deletion leads to a frame shift and early stop codons. Gene exons were flanked with loxP sites in vivo. The various mouse lines obtained were backcrossed in a similar manner and the wild-type (WT) animals used were littermates of the animal knock-outs (KO) used in each specific experiment. All mice were crossed on a C57BL/6 background for at least three generations.

1. **Echocardiogram and electron microscopy**

Echocardiography was performed in all mice after the reperfusion according to our previous studies^2^. An echocardiogram (14.0 MHz, Sequoia C512; Acuson, Germany) was used to detect both 2-dimensional and M-mode images.

Electron microscopy was conducted as previously described. The whole heart was immediately ﬁxed at 4°C with 2% glutaraldehyde in a 0.1 mol/L sodium cacodylate buffer and postﬁxed for 1 hour on ice with 1% osmium tetroxide after IR injury. After making the slices, the samples were stained with lead citrate and uranyl acetate and observed under a Hitachi H600 Electron Microscope (Hitachi, Japan)^3^. At least 30 cells in a minimum of 5 randomly selected ﬁelds were observed.

1. **Cell shortening/relengthening assay and cellular viability detection**

Mechanical properties of cardiomyocytes were assessed using a SoftEdge MyoCam system (IonOptix, Milton, MA) as our previously reported^4^. Cell shortening and relengthening were assessed using the following indices: resting cell length, peak shortening (PS), time-to-PS (TPS), time-to-90% relengthening (TR_90_), and maximal velocity of shortening/relengthening (±dL/dt).

Cellular viability was detected via caspase3 and caspase9 activity as well as MMT assay. Caspase9 and caspase3 activities were determined with caspase assay kits (Beyotime, China), which detect the production of the chromophore *p*-nitroanilide after its cleavage from the peptide substrate DEVD-*p*-nitroanilide and LEHD- *p*-nitroanilide. The MMT assay was conducted as our previously reported^5^.

1. **Terminal deoxynucleotidyl transferase dUTP nick-end labeling (TUNEL) assay and ATP detection**

The TUNEL assay was used to detect cellular death following the IR injury. For the quantiﬁcation, the numbers of TUNEL-positive cells in the infarcted areas or cardiomyocytes were calculated by counting at least 5 random separate ﬁelds as the ratio of the experimental samples to the control samples. The cellular ATP levels were measured using a ﬁreﬂy luciferase-based ATP assay kit (Beyotime) based on a ﬂuorescence technique (Genmed Scientiﬁcs Inc.) as previously described^6^.

1. **Mitochondrial respiratory assays**

Mitochondrial respiration was initiated by adding glutamate/malate to a ﬁnal concentration of 5 and 2.5 mmol/L, respectively. State 3 respiration was initiated by adding ADP (150 nmol/L); state 4 was measured as the rate of oxygen consumption after ADP phosphorylation. The respiratory control ratio (state 3/state 4) and the ADP/O ratio (number of nmol ADP phosphorylated to atoms of oxygen consumed) were calculated as previously described^2, 7^.

1. **Western blotting**

After treated, samples were washed with cold PBS and lysed with RIPA buffer containing protease inhibitor cocktail (Thermo Fisher Scientific, Waltham, MA). Lysates were centrifuged at 14,000 g for 15 min at 4˚C. Protein concentration was quantified with Pierce BCA Protein Assay Kit (Thermo Fisher Scientific, Waltham, MA). The protein extracts were separated by SDS-PAGE and electrotransferred onto PVDF membranes. Membranes were blocked with 5% fat-free milk in TBS-T buffer for 90 min and incubated overnight at 4˚C with primary antibodies. The primary antibodies for the blots were as follows: Drp1 (1:1000, Abcam, #ab56788), LC3II (1:1000, Cell Signaling Technology, #3868), Tom20 (1:1000, Abcam, #ab186734), Tim23 (1:1000, Santa Cruz Biotechnology, #sc-13298), p62 (1:1000, Cell Signaling Technology, #5114), Bcl2 (1:1000, Cell Signaling Technology, #3498), Bax (1:1000, Cell Signaling Technology, #2772), caspase9 (1:1000, Cell Signaling Technology, #9504), pro-caspase3 (1:1000, Abcam, #ab13847), cleaved caspase3 (1:1000, Abcam, #ab49822), c-IAP (1:1000, Cell Signaling Technology, #4952), survivin (1:1000, Cell Signaling Technology, #2808), Fis1 (1:1000, Abcam, #ab71498), Opa1 (1:1000, Abcam, #ab42364), Mfn1 (1:1000, Abcam, #ab57602), VDAC (1:1000, Abcam, # ab14734), CK2α (1:1000, Cell Signaling Technology, #2656). The phosphorylated-FUNDC1 (1:500) and total-FUNDC1 (1:1000) polyclonal antibodies were produced by immunizing rabbits with synthesized and purified phosphorylated and nonphosphorylated peptides from FUNDC1 (Abgent, SuZhou, China) according to our previous study^2, 8^. Representative blots were shown from three times experiments and the images were taken with an enhanced chemiluminescence (ECL) reagent.

1. **Immunofluorescence and ROS measurement**

For immunofluorescence, samples were ﬁxed with 4% paraformaldehyde for 10 min, permeabilized with 0.3% Triton X-100 for 5 min, and blocked with 10% goat serum albumin for 1 h at room temperature. Specimens were subsequently incubated with primary antibodies overnight at 4°C, washed with PBS three times and incubated with secondary antibody for 45 min at room temperature. The primary antibodies for immunofluorescence staining were as follows: cyt-c, (1:500, Abcam, #ab133504) and troponin T (1:500, Abcam, #ab8295). The mitochondrial antibody (Tom20, 1:500, Abcam, #ab186734) and lysosome antibody (Lamp1, 1:500, Abcam, #ab24170) were used to marker the mitochondria and lysosome, respectively.

Reactive Oxygen Species (ROS), a characterization of oxidant status, is involved in the injury of cells in the setting of oxidation stress. DCFH-DA (Invitrogen, Germany) was used to detect the intracellular ROS and 10 μM DCFH-DA was added to the cells culture medium, which was then incubated in the dark and measured via flow cytometry.

1. **The qPCR assay**

Total RNA was extracted from the cells using TRIzol ® reagent (Invitrogen Life Technologies, Carlsbad, CA, USA) and was reverse transcribed into a total of 1 µl (60 ng/µl) cDNA using a One‑Step RT‑PCR kit (TransGen Biotech Co., Ltd., Beijing, China), according to our previous study^9^. Quantification of gene expression was performed using an ABI PRISM 7500 Sequence Detection system (Applied Biosystems Life Technologies, Foster City, CA) with SYBR® Green (TransGen Biotech Co., Ltd.). The relative mRNA expression levels were normalized to that of β-actin using the 2^−ΔΔCT^ method. The primer sequences were as follows: CK2α (Forward Prime 5′-GGTTGTATGCTGGCAAGTATGA-3′, Reverse Prime 5′-TCGAGAGTGTCTGCCCAAGAT-3′), PGC1a (Forward Prime 5′-TCACCCTCTGGCCTGACAAATCTT-3′, Reverse Prime 5′-TTTGATGGGCTACCCACAGTGCT-3′), NRF1 (Forward Prime 5′-GGTGCCTAGTGAGAGTGAGTC CCCC-3′, Reverse Prime 5′-TCGGGGCTGAAGAGGGAGAAGTC-3′), TFAM (Forward Prime 5′-AAGGGAATGGGAAAGGTAGA-3′, Reverse Prime 5′- AACAGGACATGGAAAGCAGAT-3′).

1. **The mtDNA copy numbers and transcription level detection**

The mtDNA and nuclear amplicons were generated from a complex IV segment and GAPDH segment, respectively. The mtDNA primers were 5’-CCCCTGCTATAACCCAATACA-3’ and 5’-CCAAACCCTGGAAGAATTAAGA-3’ (fragment length, 238 bp). The GAPDH primers, chosen as the internal standards, were 5’-TGTTGCTGTAGCCATATTCATTGT-3’ and 5’-CCATTCTTCCACCTTTGATGCT-3’ (fragment length, 98 bp).

The transcript level of mtDNA was reﬂected by two different components: NADH dehydrogenase subunit 1 (ND1), which is encoded by the light chain of mtDNA, and cytochrome c oxidase subunit I (COX1), which is encoded by the heavy chain. The primers for COX1 were 5’-CCCCTGCTATAACCCAATACA-3’ and 5’-CCAAACCCTGGAAGAATTAAGA-3’ (fragment length, 238 bp). The primers for ND1 were 5’-CGGCTCCTTCTCCCTACAA-3’ and 5’-ATGGTCCTGCGGCGTATT-3’ (fragment length, 188 bp). GAPDH was selected as the internal standard. The experiments were repeated 3 times with triplicates of each sample.

**References:**

1. Rebholz H, Zhou M, Nairn AC, Greengard P, Flajolet M. Selective knockout of the casein kinase 2 in d1 medium spiny neurons controls dopaminergic function. *Biol Psychiatry* 2013, **74**(2)**:** 113-121.

2. Zhou H, Li D, Zhu P, Hu S, Hu N, Ma S*, et al.* Melatonin suppresses platelet activation and function against cardiac ischemia/reperfusion injury via PPARgamma/FUNDC1/mitophagy pathways. *J Pineal Res* 2017.

3. Winiarska K, Dzik JM, Labudda M, Focht D, Sierakowski B, Owczarek A*, et al.* Melatonin nephroprotective action in Zucker diabetic fatty rats involves its inhibitory effect on NADPH oxidase. *J Pineal Res* 2016, **60**(1)**:** 109-117.

4. Hu N, Han X, Lane EK, Gao F, Zhang Y, Ren J. Cardiac-specific overexpression of metallothionein rescues against cigarette smoking exposure-induced myocardial contractile and mitochondrial damage. *PLoS One* 2013, **8**(2)**:** e57151.

5. Zhu H, Jin Q, Li Y, Ma Q, Wang J, Li D*, et al.* Melatonin protected cardiac microvascular endothelial cells against oxidative stress injury via suppression of IP3R-[Ca2+]c/VDAC-[Ca2+]m axis by activation of MAPK/ERK signaling pathway. *Cell Stress Chaperones* 2017.

6. Chen W, Zou P, Zhao Z, Chen X, Fan X, Vinothkumar R*, et al.* Synergistic antitumor activity of rapamycin and EF24 via increasing ROS for the treatment of gastric cancer. *Redox Biol* 2016, **10:** 78-89.

7. Zhou H, Hu S, Jin Q, Shi C, Zhang Y, Zhu P*, et al.* Mff-Dependent Mitochondrial Fission Contributes to the Pathogenesis of Cardiac Microvasculature Ischemia/Reperfusion Injury via Induction of mROS-Mediated Cardiolipin Oxidation and HK2/VDAC1 Disassociation-Involved mPTP Opening. *J Am Heart Assoc* 2017, **6**(3).

8. Zhou H, Zhu P, Guo J, Hu N, Wang S, Li D*, et al.* Ripk3 induces mitochondrial apoptosis via inhibition of FUNDC1 mitophagy in cardiac IR injury. *Redox Biol* 2017, **13:** 498-507.

9. Zhou H, Yang J, Xin T, Zhang T, Hu S, Zhou S*, et al.* Exendin-4 enhances the migration of adipose-derived stem cells to neonatal rat ventricular cardiomyocyte-derived conditioned medium via the phosphoinositide 3-kinase/Akt-stromal cell-derived factor-1alpha/CXC chemokine receptor 4 pathway. *Mol Med Rep* 2015, **11**(6)**:** 4063-4072.
